# Extended two-stage designs for environmental research

**DOI:** 10.1186/s12940-022-00853-z

**Published:** 2022-04-19

**Authors:** Francesco Sera, Antonio Gasparrini

**Affiliations:** 1 Department of Statistics, Computer Science and Applications “G. Parenti”, University of Florence, Florence, Italy; 2 Department of Public Health, Environments and Society, London School of Hygiene & Tropical Medicine, London, UK; 3 Centre On Climate Change and Planetary Health, London School of Hygiene & Tropical Medicine, London, UK; 4 Centre for Statistical Modelling, London School of Hygiene & Tropical Medicine, London, UK

**Keywords:** Environmental epidemiology, Two-stage design, Meta-analysis, Temperature, Pollution

## Abstract

**Background:**

The two-stage design has become a standard tool in environmental epidemiology to model multi-location data. However, its standard form is rather inflexible and poses important limitations for modelling complex risks associated with environmental factors. In this contribution, we illustrate multiple design extensions of the classical two-stage method, all implemented within a unified analytic framework.

**Methods:**

We extended standard two-stage meta-analytic models along the lines of linear mixed-effects models, by allowing location-specific estimates to be pooled through flexible fixed and random-effects structures. This permits the analysis of associations characterised by combinations of multivariate outcomes, hierarchical geographical structures, repeated measures, and/or longitudinal settings. The analytic framework and inferential procedures are implemented in the R package mixmeta.

**Results:**

The design extensions are illustrated in examples using multi-city time series data collected as part of the National Morbidity, Mortality and Air Pollution Study (NMMAPS). Specifically, four case studies demonstrate applications for modelling complex associations with air pollution and temperature, including non-linear exposure–response relationships, effects clustered at multiple geographical levels, differential risks by age, and effect modification by air conditioning in a longitudinal analysis.

**Conclusions:**

The definition of several design extensions of the classical two-stage design within a unified framework, along with its implementation in freely-available software, will provide researchers with a flexible tool to address novel research questions in two-stage analyses of environmental health risks.

**Supplementary Information:**

The online version contains supplementary material available at 10.1186/s12940-022-00853-z.

## Introduction

In environmental epidemiological studies, it is common practice to investigate short-term associations between environmental exposures and health outcomes by analysing data collected from multiple locations. An analytical approach applied in this setting is based on the two-stage design, which has become the standard method for the analysis of multi-location data [1–12]. The design is based on the separation of the analysis into two steps: in the first stage, location-specific exposure–response associations are estimated while adjusting for various confounders; then, in the second stage, the estimates are pooled using meta-analytic methods, which can potentially incorporate location-specific meta-predictors.

The two-stage design offers several advantages. First, the pooling of data collected in multiple locations increases the statistical power, thus facilitating the detection of small risks usually associated with environmental stressors [13]. At the same time, the separation in two steps provides a flexible and computationally efficient analytical framework compared to one-stage approaches [2, 14, 15]. This allows analyses of large datasets collected across multiple populations, increasing the representativeness of the findings. Finally, an important advantage of the two-stage design is the enhanced ability to examine heterogeneity in risk across populations, which can be linked to contextual characteristics.

However, there are known limitations of this analytical method. For instance, the standard two-stage design requires the association of interest to be represented by a single effect summary (*e.g.*, a relative risk or odds ratio) for being pooled in the second stage. However, in the context of modelling exposure–response associations, this step requires the simplification of potentially complex relationships and/or the adoption of strong functional assumptions (*e.g.*, linearity). Similarly, this restriction prevents combining multiple estimates of the association of interest from the same location, for example when collected from different age groups or periods. Finally, the standard two-stage analytic design does not take into account potential geographical dependencies, often occurring in the presence of clustering. These limitations represent important barriers to the application of the two-stage framework for addressing more complex research questions about environmental health risks.

In this contribution, we illustrate a unified framework that combines multiple design extensions of the classical two-stage method for environmental health studies, some of which were described independently in published analyses [6, 16–19]. This extended two-stage framework is based on linear mixed-effects meta-analytical models, previously developed and published by our research group [20], that can combine multivariate outcomes, longitudinal settings, multilevel structures, and/or repeated measurement [20]. This framework relaxes the constraints described above and offers a flexible and generally applicable tool to implement more advanced study designs using multi-location data.

The article is organized as follows. Firstly, we introduce the extended two-stage design and its features, including the design structure and related modelling framework. Then, after presenting the specific example and the related dataset, we will demonstrate applications of the various design extensions in multiple case studies using multi-location analyses of health risks of temperature and air pollution. In a final discussion section, we describe the epidemiological context, strengths and limitations, and area of further research. Notes, data, and R scripts for reproducing the examples are added as supplementary material, with an up-to-date version available on the GitHub pages of the first and last authors.

## Methods

### Extended two-stage design

In the classical two-stage design, the data are organised and analysed in first-stage models that provide independent estimates of a single parameter representing the association of interest in each study area, for instance, a city. These effect summaries are then pooled in the second stage using meta-analytic techniques to combine the information and compute an overall estimate. As discussed above, these requirements pose important analytical constraints. The extended two-stage described here overcomes these limitations, first allowing different estimates of single or multiple parameters to be computed in each location, and then relaxing the assumption of independence of estimates within and between locations.

This extended framework provides a flexible setting that allows designing more complex epidemiological studies to address more elaborated research questions. For example, in each study area, multiple parameters could be used to represent complex exposure–response dependencies, such as non-linear and lagged temperature-health associations of temperature [21], or alternatively correlated effects of multiple exposures, such as different pollutants included in the same first-stage model [22]. At the same time, relaxing the independence assumption allows accounting for correlations arising when the locations are nested within higher geographical levels (*e.g.*, cities within countries), therefore modelling patterns of similarities and differences [19]. Moreover, in each study area, the first-stage model can be applied multiple times to obtain repeated measures of the same association, for instance longitudinally at different times or for different sub-groups, such as by age or sex. This structure allows the investigation of temporal variations in risk [17] and the flexible pooling of effect modifications [16].

These analytic features, namely complex multivariate exposure–response relationships, geographical hierarchies, and longitudinal or repeated-measure structures can be incorporated individually or simultaneously in the extended two-stage framework, offering a flexible analytic context for modern environmental research studies.

### Statistical framework

The extension of the two-stage design is made possible by the development of a unified statistical framework, previously developed and published by our research group [20], that specifies the second-stage meta-analysis as a mixed-effects linear model [20], as described below. Here we assume that estimates of the association of interest ${\widehat{{\varvec{\theta}}}}_{i}$ have been obtained from each of the $i=1,\dots ,n$ locations. Here ${\widehat{{\varvec{\theta}}}}_{i}$ generally represents the output of the first-stage analysis (see appendix A), and it can include single or multiple coefficients obtained by single or repeated measurements across times or groups, depending on the specific application. In addition, without loss of generality, such estimates can be obtained from various types of first-stage models, such as time series for aggregated data [23] or survival analysis of individual-level records [24], among others.

The first-stage estimates ${\widehat{{\varvec{\theta}}}}_{i}$ can be combined in the second stage using an extended random-effects meta-analysis that flexibly models potentially complex dependence structures. This extended meta-analytical model can be written as a linear mixed-effects model:

1 $${\widehat{{\varvec{\theta}}}}_{i}={{\varvec{X}}}_{i}{\varvec{\beta}}+{{\varvec{Z}}}_{i}{{\varvec{b}}}_{i}+{{\varvec{\varepsilon}}}_{i}$$

with $${{\varvec{b}}}_{i}\sim N\left(0,{\varvec{\Psi}}\right)$$, and $${{\varvec{\varepsilon}}}_{i}\sim N\left(0,{{\varvec{S}}}_{i}\right)$$.

The design matrix ${\varvec{X}}_{i}$, potentially expanded to account for multivariate outcomes, includes fixed-effect predictors and associated coefficients ${\varvec{\beta}}$. Random terms are represented by the design matrix ${{\varvec{Z}}}_{i}$ with coefficients ${{\varvec{b}}}_{i}$, and by the errors ${{\varvec{\varepsilon}}}_{i}$. The random terms have (co)variance matrices ${\varvec{\Psi}}$ and ${{\varvec{S}}}_{i}$, representing the deviations and errors between and within locations, respectively.

It is important to note that the association parameter ${\widehat{{\varvec{\theta}}}}_{i}$ could have a general nested design with *L* level inducing possible non-independence of the estimates, e.g. associations estimated at multiple times, or in cities nested within a country. The extended framework naturally considers the nested design with a hierarchy of the random-effects effects vector${{\varvec{b}}}_{i}$, then ${{\varvec{b}}}_{i}$ consists of the random coefficients operating on the levels (from outer to inner)$l=1,\dots ,L$: ${{\varvec{b}}}_{i}^{T}=\left({{\varvec{b}}}_{i1}^{T},\dots ,{b}_{iL}^{T}\right)$, and the design matrix ${\mathbf{Z}}_{i}$ of the random terms has the corresponding partitioning${{\varvec{Z}}}_{i}=\left({{\varvec{Z}}}_{i1}|\dots |{{\varvec{Z}}}_{iL}\right)$,${{\varvec{Z}}}_{il}=\left({{\varvec{Z}}}_{il1}|\dots |{{\varvec{Z}}}_{ilnl}\right)$. Note that every matrix ${{\varvec{Z}}}_{ilj}$ has nonzero entries only in the rows that correspond to units in the group *j* $(j=1,\dots ,{n}_{l})$ of level$l$.

The (co)variance matrix of the random terms has then the following structure:

$${\varvec{\Psi}}=\sum_{j=1}^{{n}_{l}}{{\varvec{Z}}}_{ilj}{{\varvec{\Psi}}}_{l}{{\varvec{Z}}}_{ijl}^{T}$$

where ${{\varvec{\Psi}}}_{l}$ is the covariance of the random terms operating at level $l$.

### Example and data

The various extensions of the two-stage design will be illustrated using the same analytical example of multi-city time-series data collected as part of the National Morbidity, Mortality and Air Pollution Study (NMMAPS) [25]. This database contains, among other information, daily series of mortality counts and weather and pollution measurements totalling 5114 observations for the period 1987–2000 in each of 108 cities in the USA. This data resource has been used in several epidemiological analyses to assess health risks associated with air pollution and later with temperature [5, 26–30].

The NMMAPS data consisted of daily series of all-cause and cause-specific mortality, also stratified by age groups (0–64, 65–74, 65 and older), and various indices of daily levels of several pollutants and weather variables. In addition, the database included city-level metadata with several variables on geographical, climatological, demographic and socio-economic characteristics. The original datasets were collected on the 15th of May, 2013 through the package NMMAPSdata in the R software [31]. The package is now archived and the mortality series are not provided anymore. The data are here complemented with information on air conditioning use, collected longitudinally for a subset of cities and obtained from different sources [17].

The database is used in a series of case studies described in the next sections to illustrate the various extensions of the two-stage design. In each of them, we assume that first-stage models have been performed separately in the 108 locations, collecting summary estimates of association parameter(s) ${\widehat{{\varvec{\theta}}}}_{i}$ and their (co)variance matrix $V\left({\widehat{{\varvec{\theta}}}}_{i}\right)$, and optionally location-specific metadata. These summaries are made available in the Supplementary Material, together with the R code for the first-stage step to produce these quantities from the original data, and the R code and data for the second-stage step to reproduce the results of the case studies. Methodological and analytical details, in particular related to the first-stage modelling, are omitted to focus on specific aspects of the extensions of the two-stage design, with additional information provided in the Supplementary Material. As methodological case studies, these analyses should be considered illustrative examples and are not meant to offer substantive epidemiological evidence.

## Results

### Case study 1: modelling complex multi-parameter associations

#### Motivation

As mentioned earlier, an important limitation of the standard two-stage design is the need to simplify the relationship estimated in the first stage in a single effect summary, for it to be pooled in the second stage. This prevents the modelling of more complex associations represented by multiple parameters.

This limitation can be addressed by extending the two-stage design so that multiple quantities can be jointly combined in the second stage, using meta-analytic models that take into account their multivariate structure and their covariance (correlation) within and between locations. The meta-analytical methods can be further extended to multivariate meta-regression models that include specific predictors to explain (part of) the observed heterogeneity. This extension of the two stage design has been known as a multivariate meta-analysis or multivariate meta-regression [15], and it can be represented as a specific parametrisation of the linear mixed effects meta-analytic framework presented above. These extensions can be implemented with the R package mvmeta [32] or with the updated and more general R package mixmeta [33].

In this case study, we offer an example of this extension to assess health risks associated with outdoor temperature, often characterised by marked non-linearity and heterogeneity of the effects across locations. In particular, we will investigate the association between heat and all-cause mortality during the summer months and the potential role of city-specific characteristics in modifying the risk. This extension of the two-stage design has been previously used in published analyses which evaluated the short-term health impacts of temperature [6, 16, 34].

#### Brief description of the data, model, and analysis

We assume that summer-only time series models have been fitted in each of the 108 NMMAPS cities to estimates temperature-mortality relationships using spline functions (see Supplementary Material B1), obtaining sets of four coefficients and their (co)variance matrices that represent the multi-parameter non-linear associations. In the second stage, we use these estimates as multivariate outcomes in the extended meta-analytical framework.

First, we fit a multivariate meta-analysis using a maximum likelihood (ML) estimator to pool the first-stage results and obtain an estimate of the average heat-mortality exposure–response curve. We then attempt to identify possible contextual characteristics that explain a quota of heterogeneity. Among potential factors, we consider population size, education (% of people with high-school degree) and unemployment (% of unemployed). These variables are included as predictors in multivariate meta-regressions, and their effects tested through likelihood ratio (LR) tests. Finally, a stepwise procedure is applied to select the best set among univariable and multivariable models. See Supplementary Material B1 for details.

#### Results

**Fig. 1:**
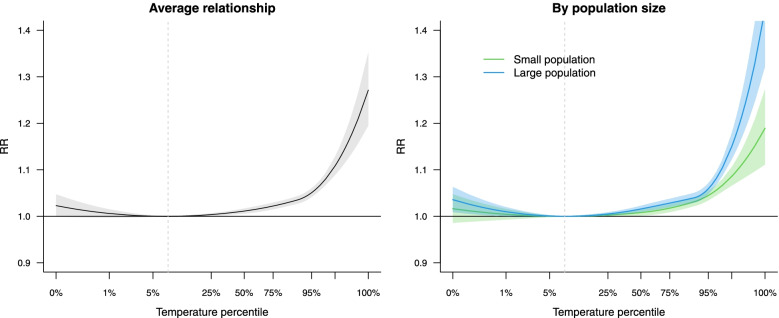
Pooled association between relative temperature (percentiles) and all-cause mortality in 108 US cities during the summer period in 1987–2000 in Case Study 1. The x-axis is scaled so that the summer temperature distribution match the average percentiles of all the cities. The left panel shows the average heat-mortality curve estimated by the multivariate meta-analysis. The right panel illustrate the effect modification from population size, predicted from the full multivariate-meta-regression at the 10^th^-90^th^ percentile values of the city-specific meta-variable

The basic multivariate meta-analytic model (with no predictors and only intercepts) produces pooled estimates of the set of coefficients representing the average heat-mortality association across the 108 cities. These coefficients can be used to compute the non-linear exposure–response curve expressed as relative risk (RR) by applying the same spline transformations on an average summer temperature distribution represented in a relative percentile scale [15]. The results are displayed in Fig. 1, showing a minimum mortality risk at low summer temperatures (MMT) and the sharp increase of the RR beyond the 90^th^ percentile.

The simple meta-analysis shows a substantial heterogeneity in heat-mortality associations across cities, with an I^2^ of 61.5% and a highly significant Cochran Q test (*p*-value < 0.001). Therefore, we assess if some of this heterogeneity was explained by some city characteristics, specifically population size, education, and unemployment, by adding them as predictors in multivariate meta-regressions. Results are reported in Table 1. When tested separately in univariable models, each predictor is significantly associated with modification of the heat-mortality association. The full multivariable model identifies instead independent associations only for population size and unemployment, and these results are consistent with the selection of the forward stepwise procedure.

**Table 1:** Degrees of freedom (df), I^2^, information criteria, and likelihood ratio (LR) tests for meta-predictors in second-stage multivariate regression models of Case Study 1. The last model selected by forward stepwise procedure includes only population size and unemployment

		df	I^2^ (%)	AIC	BIC	LR test (*p*-value)
Model 0	Intercepts	14	61.5	-520.60	-463.64	
Model 1	+ population size	18	53.3	-529.81	-456.57	0.002
Model 2	+ education	18	58.1	-530.26	-456.80	0.002
Model 3	+ unemployment	18	55.7	-536.24	-463.11	< 0.0001
Model 4	Full model	26	48.3	-539.60	-433.82	
Model 5	Stepwise-selected model	22	49.7	-543.67	-454.16	

The tests above demonstrate an effect modification by specific city-level meta-variables, but provide little information on its direction. This can be identified by using the parameters of the multivariate meta-regression models to predict the multivariate outcome, namely the coefficients of the spline function representing the heat-mortality relationship, for given values of the meta-predictors. As an example, we used this method to isolate the effect modification of population size, keeping the other meta-predictors constant. The results, shown in the right panel of Fig. 1, indicate a higher mortality risk of heat in larger cities.

This case study demonstrates an extension of the two-stage design to pool multi-parameter associations. The specific example illustrates an application for complex exposure–response relationships, but the multi-parameter definition can be generalised, and the method is applicable for instance also to pool effects of multiple pollutants or multiple health outcomes [22].

### Case study 2: modelling complex hierarchical structures

#### Motivation

Another important limitation of the standard two-stage design is the assumption of conditional independence between first-stage estimates. In environmental epidemiological associations, this assumption is invalid in the presence of geographical clustering, occurring when estimates are more similar in locations within the same region than between regions.

The two-stage design can be extended accordingly by modelling the dependencies among estimates through a hierarchical structure (*e.g.*, cities within countries, or countries within states). This extension can be implemented through a second-stage multilevel meta-analysis that defines multiple sets of random effects at different geographical levels.

In this case study, we provide an example in an analysis of the association between air pollution and non-accidental mortality in a multi-city time series study. Specifically, we assess the increased risk associated with exposure to ozone in a sample of NMMAPS cities accounting for clustering within states. We previously applied this extended two-stage design in a study evaluating the short-term health effects of pollutants [19].

#### Brief description of the model, data, and analysis

As in the previous case study, we assume that first-stage time series models have been performed in each city, collecting estimates of the log-RR for an increase in ozone of 10 µg/m^3^, along with its variance as a measure of the uncertainty (see Supplementary material B2). Estimates for cities with no or limited daily measurements of ozone were set to missing, leaving a sample of 98 cities within 38 states.

We start the analysis by fitting a standard meta-analysis with city-specific random effects. Then, in order to account for potential geographical differences, we first perform a standard meta-regression with state indicators as fixed-effects predictors, and then the extended model including two levels of random effects by cities nested within states. Finally, we compute state-level fixed-effects predictions from the meta-regression, and best linear unbiased predictions at both city and state level from the multilevel model [20]. See Supplementary material B2 for details.

#### Results

The standard meta-analytic model with single-level random effects for cities returns a pooled RR of non-accidental mortality of 1.0037 (95%CI: 1.0027 to 1.0047), corresponding to a percentage increase of 0.37%, with a between-city variance equal to 0.0049^2^. The inclusion of state indicators in the meta-regression suggests that there are significant geographical differences (LR test with a *p*-value < 0.001). Two drawbacks of this fixed-effects approach are the lack of a pooled effect estimate, and the high uncertainty in state-level predictions given the low number of cities within states and an highly-parameterised model.

**Fig. 2:**
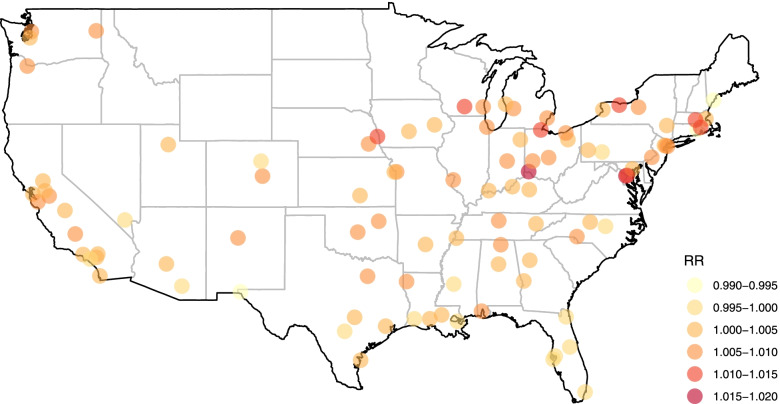
City-level best linear unbiased predictions of the RR of non-accidental mortality for 10 µg/m^3^ increase in ozone in 97 US cities (Honolulu not shown) during 1987–2000, as computed from the two-level random-effects meta-analysis in Case Study 2

The multilevel random-effects model addresses these limitations. First, this model provides a pooled relative risk of 1.0038 (95%CI: 1.0024 to 1.0051), with a similar point estimate and slighter higher confidence intervals than the standard meta-analysis. The between-group heterogeneity is split between states (0.0030^2^) and cities (0.0040^2^), suggesting variation at both levels. Figure 2 displays these geographical differences by mapping the city-level best linear unbiased predictions (BLUPs) of the RR for a 10 µg/m^3^ increase in ozone.

**Fig. 3:**
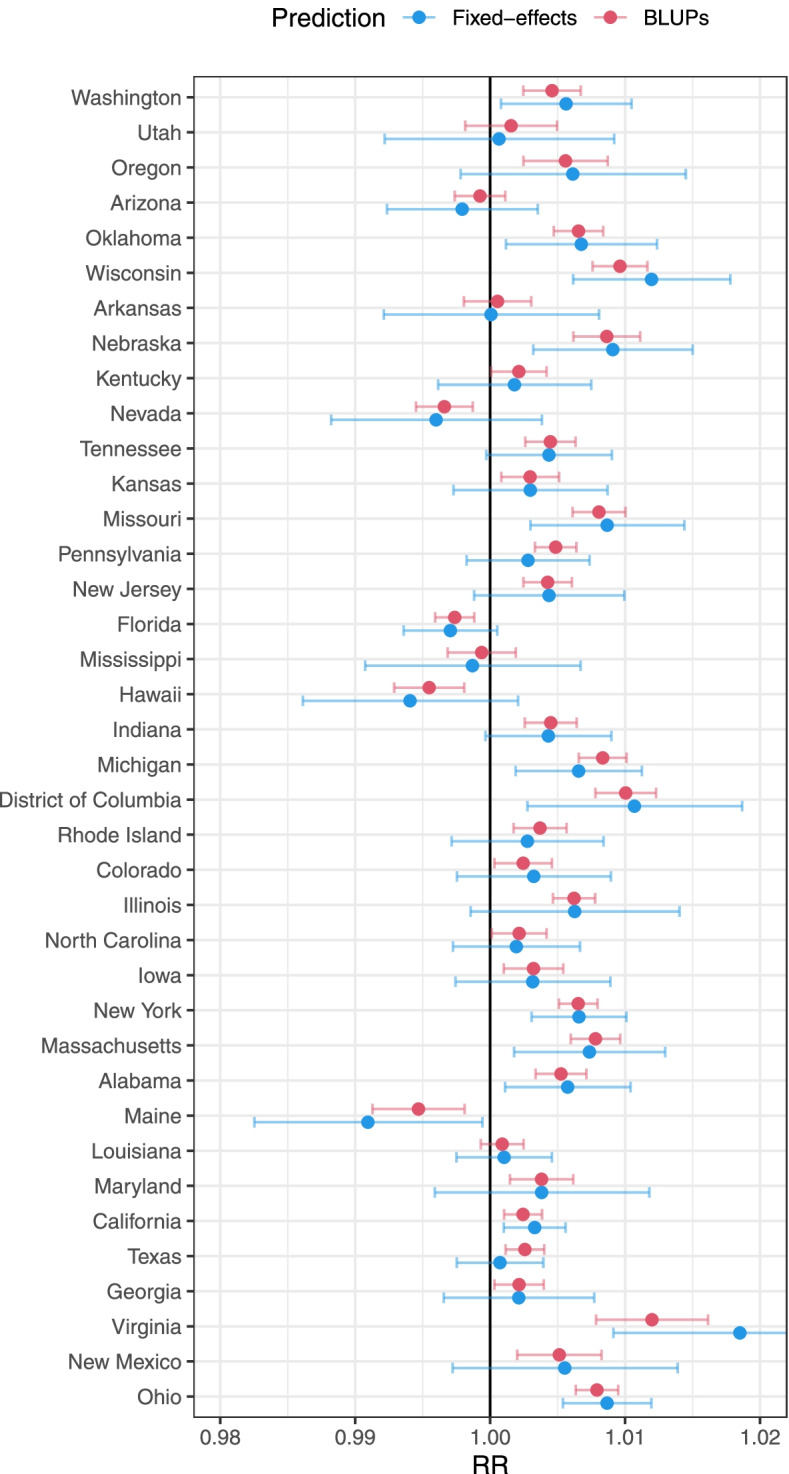
Relative risk (RR) of non-accidental mortality for a 10 µg/m^3^ increase in ozone across US states during 1987–2000 in Case Study 2. Estimates were obtained as state-level fixed-effects predictions from a standard meta-regression model (blue) and as best linear unbiased predictions (BLUPs) from a two-level random-effects model (red)

Second, the multilevel model can improve the state-specific estimates by computing BLUPs at this geographical level. Figure 3 compares these quantities with fixed-effects predictions obtained from the standard meta-regression model. The results reveal the gain in precision of the BLUPs resulting from the shrinkage and borrowing of information across states [20]. These estimates are more reliable than fixed-effects predictions, where only the within-state information is used.

This case study illustrates how to extend the classical two-stage design by accounting for hierarchical dependencies between estimates from different locations. This flexible multilevel structure offers the possibility to separate the heterogeneity across geographical levels and to obtain more reliable and informative association estimates. The approach can be seamlessly extended to multi-parameter associations, combining multilevel and multivariate models [18].

### Case study 3: sub-groups analysis, and dose–response relationships

#### Motivation

Common applications of the two-stage design entail the provision of single effect summaries from each location. However, the analysis can sometimes be repeated by sub-groups of the population defined by specific characteristics, such as sex or age, resulting in repeated measures and dependencies that the standard two-stage design is not able to handle.

The extended framework addresses this limitation, offering an adaptable grouping structure that allows multiple association estimates within a location. Moreover, the role of sub-groups characteristics can be flexibly examined in a dose–response fashion by including either categorical and continuous variables in the fixed-part component. As for the extensions presented in the previous case studies, this framework is also applicable to multivariate outcomes.

In this case study, we extend further the investigation of the association between heat and all-cause mortality illustrated in Case Study 1 by stratifying the analysis by age. This provides repeated estimates for each of the 108 NMMAPS cities and the opportunity to apply flexible models to examine patterns of risk varying by age.

#### Brief description of the model, data, and analysis

The stratified analysis involves the fitting of the same first-stage regression model as in Case Study 1, but this time repeated separately for the three age groups (0–64, 65–74, 65 and older) using age-specific mortality series (see Supplementary material B3). We assume that this step has been performed and that we have obtained 324 sets of coefficients and associated (co)variance matrices representing age-specific heat-mortality associations in three age groups and 108 cities.

In the second stage, we first fit a standard meta-regression that ignores the city-level clustering and models the 324 multivariate outcomes using categorical indicators for age groups and unit-specific random effects. This model is first extended to account for clustering by defining the random-effect grouping structure at the city level. Then, we specify a continuous age variable by assigning specific values to the groups (60, 70, and 85 years) and finally we model it using either a linear or non-linear spline parametrisation. See Supplementary Material B3 for details.

#### Results

Table 2 offer a comparison between the different modelling strategies. All the models indicate evidence for an effect modification of age, but those correctly accounting for clustering by defining city-level groups (Models 1–3) demonstrate a better fit. The comparison of the more flexible models that define a continuous dose–response parametrisation (Models 2 and 3) suggests the presence of non-linearity. Note that the spline model (Model 3) has virtually an identical fit of the model with categorical indicators (Model 1), given that the number of groups/values equals the spline terms. However, the more flexible option defining the effect modification on a continuous scale has still some advantages, as illustrated below.`

**Table 2:** Comparison of various second-stage repeated-measure meta-analytical models to examine age-specific associations between heat and all-cause mortality in Case Study 3. The table report if clustering is accounted for, the parametrisation of age, the I^2^ index and information criteria

	Clustering	Age parametrisation	I^2^ (%)	AIC	BIC	LR test for age (*p*-value)
Model 0	No	Categorical	36.0	-480.99	-367.82	0.004
Model 1	Yes	Categorical	36.0	-553.06	-439.38	< 0.001
Model 2	Yes	Linear	36.9	-543.27	-450.26	< 0.001
Model 3	Yes	Non-Linear	36.0	-553.06	-439.38	< 0.001

**Fig. 4:**
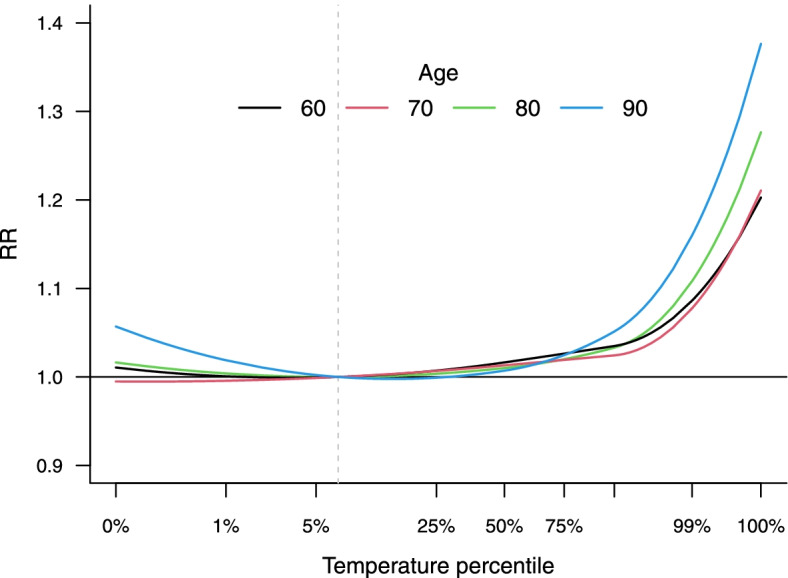
Average temperature-mortality relationships across 108 US cities during the summer period in 1987–2000 predicted at different ages (in years) from the extended model with a continuous spline parametrisation (Model 3) in Case Study 3

The analysis has similarities to Case Study 1, which illustrated the effect modification related to city-specific variables, but, in this case, modelling within-city variations in risk. Still, the direction of the effect is difficult to ascertain when applying complex multi-parameter functions. Therefore, we rely on the same approach to predict average heat-mortality exposure–response curves for specific age values, taking advantage of the continuous dose–response parametrisation of the repeated-measure multivariate model. The results are reported in Fig. 4, suggesting a clear age pattern with the risk of heat increasing at older ages.

This case study shows how to extend the classical two-stage design to account for repeated measures originating, for instance, in the presence of multiple estimates from population sub-groups in the same location. This design extension also offers the possibility of modelling effect modifications by specific characteristics using flexible dose–response parametrisations on a continuous scale. It is interesting to note that this approach relaxes the requirement of defining fixed sub-groups (e.g., by age), as different values can be attributed across locations.

### Case study 4: modelling longitudinal patterns of risk

#### Motivation

A different setting in which repeated measures can arise in two-stage analyses is when multiple estimates are collected at different times for the same location. This situation poses methodological problems that, similarly to the previous case study, standard designs are not equipped to handle.

The development of the two-stage methods to address these limitations requires accounting for the longitudinal structure of the data and modelling temporal trends in the exposure–response association. This extension provides environmental epidemiologists with the possibility of studying longitudinal patterns of risk, and considering potential time-varying factors explaining the variability of the estimated association over time.

In this case study, we again revise the analysis of heat-mortality relationships described in Case Study 1 by fitting the model in multiple sub-periods in each city. This step offers the opportunity to study temporal changes in the exposure–response curve and to assess the role of air conditioning (AC) in attenuating the risk. This case study is an illustrative example of a published analysis by our research group [17].

#### Brief description of the model, data, and analysis

We assume that in the first stage the data for the subset of 89 NMMAPS cities with information on AC data were split into five sub-periods (1987–98, 1990–92, 1993–95, 1996–98, and 1999–2000), and that separate time series models were fitted in each city/period combination, deriving a total of 445 sets of coefficients (co)variance matrices representing the multivariate association. Each city/period combination can be assigned a measure of AC prevalence use (%) reconstructed from an external database [17] (see Supplementary Material B4).

In a second step, we apply a longitudinal multivariate random-effect meta-regression to evaluate changes in heat-related mortality risks, accounting for both within and between-city variations. We include in the model a smooth spline function of calendar year and a linear term for AC as time-varying predictors, assessing their contribution with LR tests. As in the previous case study, this flexible continuous parametrisation allows the prediction of non-linear exposure–response curves for any given year and potential scenarios of AC use. See Supplementary Material B4 for details.

#### Results

**Fig. 5:**
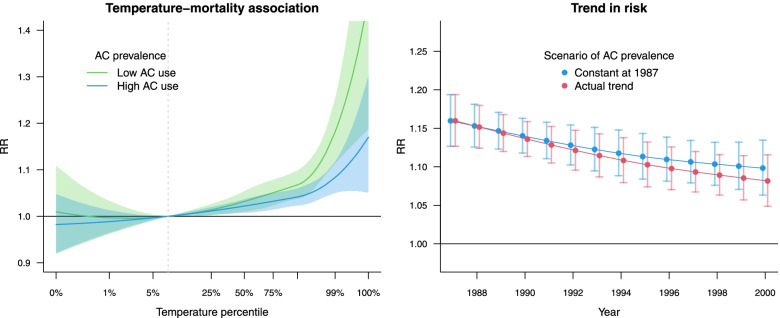
*Left panel*: predicted average heat-mortality association (in RR) during the summer predicted for different air conditioning (AC) prevalence (20% and 80%) in Case Study 4. *Right panel*: trends in RR at 99^th^ summer temperature predicted under two scenarios of AC use, corresponding to the observed average and a constant 1987 value

The longitudinal meta-regression model suggests an independent effect of both calendar year (LR test *p*-value = 0.038) and air conditioning (*p*-value = 0.008). We evaluate their role by predicting the exposure–response associations in RR scale for different AC prevalence levels (80% vs 20%) in the year 1990. The curves are displayed in Fig. 5 (left panel), indicating how increasing AC has a protective effect at hot temperatures.

In order to assess the joint contributions of trends and AC use, we depict two scenarios to represent longitudinal changes in risk along years: a factual scenario using the observed trend in average AC prevalence, and a counterfactual scenario with AC use kept constant in time at the value of 1987. The right panel of Fig. 5 shows the results, summarising the heat effects as the RR computed at the 99^th^ percentile versus the MMT along the period 1987–2000. The predicted risk under the counterfactual scenario (in blue) reveals a decreasing trend independent from AC use. Nonetheless, the comparison with the factual scenario (in red) suggests that the increase in AC prevalence during the period contributed somehow to attenuate the risk.

This last case study demonstrates the extension of the two-stage design to study longitudinal associations, evaluating changes in risk across both spatial and temporal dimensions. The flexibility of the extended framework allows parametrising effects on a continuous scale and performing second-stage meta-analysis with balanced and unbalanced data, with important design advantages.

## Discussion

In this contribution, we presented several design extensions of classical two-stage studies, and introduced several examples that illustrate how the flexibility of this modelling tool can improve the investigation of the effect of environmental exposures on health outcomes. Specifically, we showed how the extended two-stage design can be applied to investigate complex exposure–response dependencies, multilevel longitudinal structures, and repeated-measure dose–response associations. The analytic framework can be applied using classical inferential procedures and can be easily implemented using the R package mixmeta.

The two-stage design was proposed for the analysis of multi-location data. The methodology has been popularised by multi-city time series studies investigating short-term risk associations with environmental stressors [2, 5, 10], and it has become a common tool to assess the acute effects of pollutants [4, 7–9, 11] and temperature [1, 3]. The two-stage design has been also implemented in multi-cohort studies (e.g. ESCAPE project) to evaluate to long-term effect of pollutants [12, 24, 35], and in genetic epidemiology studies [36, 37]. Several extensions of a standard design have been proposed over the years, all of which can be represented as specific applications of the unified framework proposed here.

The most straightforward extension considers multiple estimates obtained in the first stage and the application of multivariate meta-analytic models in the second stage. This approach was originally developed to pool lagged effects [2], multiple pollutants [22], and non-linear dependencies [15], or more complex distributed lag non-linear associations [38].

Early applications of the two-stage design considered a small number of locations within a country, but the increased availability of environmental measures and health data now allows studies that include hundreds of locations within several countries [18, 19, 39]. In this setting, the locations can have a hierarchical structure that can be directly incorporated into the extended two-stage design. This extension has been proposed to obtain global, country, and city-level estimates of the associations by combining information within and between locations [18, 19, 39].

Environmental risk factors are often associated with risks that vary according to some individual or contextual characteristic [28, 40, 41]. The comparison of association measures across sub-groups was originally performed qualitatively and/or without consideration of the possible non-independence of multiple estimates collected within a location [42]. The extended two-stage design can directly model dependencies between the stratified estimates within each location, and appropriate inferential procedures can be used to evaluate differences across sub-group estimates.

In addition, such differences can be linked with measurable characteristics that can be included as categorical and continuous fixed-effects terms in the extended second-stage meta-regression. This extension allows modelling risks varying both within locations (*e.g.*, age in Case Study 3) and between locations (*e.g.*, population size and unemployment rate in Case Study 1). This effect modification patterns can be modelled linearly or non-linearly using flexible parametric functions, representing a further extension of dose–response pooling methods applied in observational studies [43, 44].

With the availability of longer time series of environmental exposures and health outcomes, researchers have started to investigate the temporal variation in associations of short term environmental exposures and health outcomes [3, 17, 45–49]. In particular, modelling approaches have proposed time-varying extensions of distributed lag non-linear models [47, 48], Bayesian hierarchical models [3, 46], and functional meta-regression [49]. The extended two-stage design naturally accommodates balanced and unbalanced association parameters longitudinally directly accounting for possible non-independences, and it provides the possibility to parametrise trends through linear and non-linear functions. It is important to note that the longitudinal setting can incorporate other extensions, such as multivariate outcomes and multilevel structures, modelling potentially complex structures of longitudinal associations [17].

The data example and the four case studies are consistent with the most common application of the two-stage design in time series analysis of short-term effects of environmental exposures. However, it is worth noting that the framework proposed here is not restricted to the time series setting, and first-stage estimates can be obtained by any other approach such as case-crossover or time-to-event Cox models. Therefore, the extended two-stage design can similarly be applied in environmental epidemiological studies investigating either short or long-term effects of environmental exposure, using either individual-level or aggregated cross-sectional, case–control, and cohort data [12, 24, 35–37, 50].

An important advantage of the proposed development is the fact that it is grounded on a unified likelihood-based inferential framework and implemented in freely available and easy-to-use software. All the analyses illustrated in the four case studies can be performed using the R package mixmeta, which offers a simple syntax to define all the different models and combinations of them. Similar extensions of the two-stage design were proposed based on Bayesian hierarchical models, for instance for multivariate [22], multilevel [14] and longitudinal data [46], but they usually require advanced statistical and programming skills and can be computationally more demanding. Nonetheless, the Bayesian framework offers more flexibility in accommodating random-effects and correlations, for instance spatial structures that are not yet available and generally more difficult to implement in our likelihood-based development.

## Conclusions

Technological developments in environmental monitoring, coupled with advancements in data linkage and collaborative tools, offer new opportunities for researchers to collect large multi-locations databases. The development of a general and extended framework for two-stage designs is therefore timely and offers a flexible and generally applicable tool for modern environmental epidemiological studies.

## Supplementary Information

**Additional file 1. **

## Data Availability

Data, and R scripts for reproducing the examples are added as supplementary material, with an up-to-date version available on the GitHub pages of the first and last authors.

## Abbreviations

NMMAPS: National Morbidity, Mortality and Air Pollution Study
ML: Maximum likelihood
LR: Likelihood ratio
RR: Relative risk
MMT: Minimum mortality risk temperature
BLUPs: Best linear unbiased predictions
AC: Air conditioning
AIC: Akaike information criterion
BIC: Bayesian. information criterion
