# Correction: Extended two-stage designs for environmental research

**DOI:** 10.1186/s12940-022-00861-z

**Published:** 2022-05-09

**Authors:** Francesco Sera, Antonio Gasparrini

**Affiliations:** 1 Department of Statistics, Computer Science and Applications “G. Parenti”, University of Florence, Florence, Italy; 2 Department of Public Health, Environments and Society, London School of Hygiene & Tropical Medicine, London, UK; 3 Centre on Climate Change and Planetary Health, London School of Hygiene & Tropical Medicine, London, UK; 4 Centre for Statistical Methdology, London School of Hygiene & Tropical Medicine, London, UK

**Correction to: Environ Health 21, 41 (2022)**

**https://doi.org/10.1186/s12940-022-00853-z**

The authors would like to make a correction to the article entitled “Extended two-stage designs for environmental research” published in Environmental Health in April 2022 [1]. Due to problems during the revision of the proofs, the R code and data to reproduce the examples described in the four case studies were not included in the original version of the article. The material is available in a GitHub repository, freely accessible by the readers. The following parts of the manuscript have been revised to provide this information:

- Page 2, left column, last sentence of Introduction: “Notes, data, and R scripts for reproducing the examples are added as supplementary material, with an up-to-date version available on the GitHub pages of the first and last authors”. The sentence has been changed to: “An up-to-date version of the notes, data, and R scripts for reproducing the examples are available on a GitHub repository (see Availability of data and material)”.
- Page 3, right column, last paragraph of Methods. The third sentence: “These summaries are made available in the Supplementary Material, together with the R code for the first-stage step to produce these quantities from the original data, and the R code and data for the second-stage step to reproduce the results of the case studies” has been revised to: “These data are made available in a GitHub repository, together with the R code for the first stage to produce these quantities from the original data, and for the second stage to reproduce the results of the case studies (see Availability of data and material)”.
- Section Availability of data and materials. The text in this section has been revised to: “An up-to-date version of the R scripts and data to fully reproduce the examples described in the four case studies are added in a GitHub repository, available at https://github.com/gasparrini/Extended2stage.”

This correction does not affect the results reported in the article and their interpretation. The authors apologise for the mistake.

The original article has been updated.
