# Supplementary material for: Extended two-stage designs for environmental research
Source: Environ Health. 2022 Apr 19;21:41. doi: 10.1186/s12940-022-00853-z (PMC9017054; doi:10.1186/s12940-022-00853-z)
Supplement: Supplementary file 1 — Additional file 1. [file 12940_2022_853_MOESM1_ESM.docx]

**A. First-stage time-series models**

In the examples described in the four case studies, the first-level data are time series, and were modelled accordingly. In each first-level unit *i*, a set of N*_i_* observations are defined at equally-spaced time intervals (here corresponding to days) *t*=1,…,N*_i_*. In the first stage, the aim is to estimate the association between the exposure x*_it_* and the outcome *Y_it_* after controlling for the set of the time-varying confounders **c***_it_*, in addition to time trends. Usually, this aim is achieved by fitting generalized linear models (usually with Poisson or quasi-Poisson family) (1):

| $g\left[ \text{E}\left( Y_{it} \right) \right]=\alpha+f\left( x_{it}\mathcal{,l;}\boldsymbol{\theta}_{i} \right)+\sum_{q=1}^{Q} s_{qi}\left( t;\boldsymbol{\delta}_{i} \right)+\sum_{p=1}^{P} h_{pi}\left( \boldsymbol{c}_{ipt};\boldsymbol{\gamma}_{i} \right)$ | (1) |
| --- | --- |

Where the function $f\left( x_{it}\mathcal{,l;}\boldsymbol{\theta}_{i} \right)$ specifies the association with the exposure of interest $x$, allowing non-linearity and complex temporal dependencies along the lag dimension $\mathcal{l}$. These complex relationships can be modelled through distributed lag linear and non-linear models (DLMs and DLNMs), which can flexibly define cumulative effects of multiple exposure episodes (2). The term(s) $s_{qi}$ represent functions expressed at different timescales to model temporal variations in risk associated with underlying trends or seasonality, among others.

**B. Modelling details about the four case studies**

**B1.** **Pooling complex multi-parameter associations**

In the first stage, we fitted distributed lag non-linear time-series models for each of the 108 US cities in order to estimate the association between mean temperature and overall mortality in summer months (June to September). Briefly, the bi-dimensional cross-basis function was composed of a quadratic B-spline system with two internal knots used to represent non-linearity in the temperature dimension, and an unconstrained parameterization in the lag space within 0-3 days (2). The coefficients of the cross-basis were then “reduced” over the lag dimension to obtain parameters representing the non-linear net association between mean temperature and overall mortality (2) obtaining a set of four coefficients vector ${\hat{\boldsymbol{\theta}}}_{i}$ and their (co)variance matrix $\boldsymbol{S}_{i}$ for each of the 108 cities (3). The confounding effect of long-term trends and seasonality was modelled through interaction of a smooth function of day of the year (natural splines with four degrees of freedom) and year.

In the second-stage, the extended random-effects meta-analysis can be used to obtain the pooled set of coefficients taking into account the dependencies and uncertainty of the coefficients as measured by the covariance matrix of the coefficient set for each city.

In terms of the extended framework, this represents an example of multivariate meta-analysis, where the design matrices of the fixed and random effects are identity matrices with dimensions equal to the number of coefficients: $\boldsymbol{X}_{i}=\boldsymbol{Z}_{i}=\boldsymbol{I}_{4}$. Note that the random coefficient vector $\boldsymbol{b}_{i}$ is a $(4\times1)$ row vector.

We then fitted meta-regression models to identify contextual factors that could explain a quota of the heterogeneity. Among several potential factors, we considered population size, percentage of people with high-school degree, and percentage of unemployment. These variables will be included in the fixed-effects part of the meta-analytic model by setting $\boldsymbol{X}_{i}=\boldsymbol{x}_{i}^{T}\otimes\boldsymbol{I}_{4}$ , with $\otimes$ is the Kronecker product operator, where $\boldsymbol{x}_{i}^{T}$are the variable values measured in first-level unit *i*. With this parametrisation, in equation (1), $\boldsymbol{\beta}$ is the (12 X 1) dimensional coefficient vector that defines the association of the 4 outcomes with the 3 predictors.

**B2. More complex hierarchical structure**

In the first-stage, we fitted quasi-Poisson time-series models for each of the 85 US cities with available data in order to estimate the association between ozone and non-accidental mortality. Ozone was modelled linearly over the moving average of 0-1 lags. The parameters of the DLM model were “reduced” over the lag dimension, similarly to the previous case stury, obtaining a single coefficient (log-RR for an increase of 10 µg/m3) and its variance for each of the 85 cities. We considered the confounding effect of temperature and long-term trend/seasonality. Briefly, temperature was modelled using a cross-basis composed of quadratic B-spline function with three internal knots (at the 10^th^, 75^th^, and 90^th^ percentiles) to represent non-linear exposure-response relationships, and and unconstrained parametrisation within 0-3 days to model the lag-response shape. The confounding effect of long-term trends was modelled with a smooth function of day of the year (natural splines with four 7 degrees of freedom per year).

We estimated the log-RR for an increase of 10 µg/m3 in each of the 85 cities, along with its variance as a measure of the precision (uncertainty). These estimates are nested within 35 states.

Within the extended meta-analytic framework, we structured the first stage estimated coefficients ${\hat{\boldsymbol{\theta}}}_{i}$ considering two hierarchical levels $L=2$, with cities nested within US states. Note that ${\hat{\boldsymbol{\theta}}}_{i}$ is defined at the highest hierarchical level (US states) with $n_{i}$ cities, and that cities and US states have specific random effects (e.g. random intercepts)$\boldsymbol{b}_{i1}^{T},\boldsymbol{b}_{i2}^{T}$ with design matrices $\boldsymbol{Z}_{i1}$ , a column of ones, and $\boldsymbol{Z}_{i2}$ with ones on the specific row and column identifying the city. Here $\boldsymbol{X}_{i}=\mathbf{1}$ , $\left(n_{i}\times1\right)$, a column of ones and specifies the intercept term. This is an example of multilevel meta-analytic model.

**B3. Sub-group analysis, modelling dependencies and dose-response relationship**

The NMMAPS dataset provides daily time-series of overall mortality data for three age classes: [0, 65), [65, 75), and [75+) for each of the 108 US cities. The analysis follows the same scheme as in Section B1, only repeated by each age class. Thus, we obtained 324 sets of coefficients and associated (co)variance matrix describing the age-specific temperature mortality association nested within 108 cities (i.e., each city has three coefficients).

When considering the clustering effect of cities, the first stage estimates ${\hat{\boldsymbol{\theta}}}_{i}$ is a $(12\times1)$ vector (4 splines coefficients estimated for the three age classes) and the within-location (co)variance matrix $\boldsymbol{S}_{i}=\oplus_{a}\boldsymbol{S}_{i,a}$ is a block diagonal matrix where diagonal blocks $\boldsymbol{S}_{i,a}$ are the covariance matrices of the coefficients calculated in each age class (a). We modelled age as a fixed-effects variable by stacking by rows the matrices $\boldsymbol{X}_{i,a}$, with $\boldsymbol{X}_{i,a}=\boldsymbol{x}_{i,a}^{T}\otimes\boldsymbol{I}_{4}$ , where $x_{i,a}^{T}$ is the vector representing the age class (a) in the different parametrisations: (categorical; 2 dummies variables), (linear; 1 variable), (non-linear; natural splines with two degrees of freedom). Note that in this case the parametrisation of age as categorical and non-linear with the chosen cut-offs of 70 years are equivalent, as there are three age groups. However, the two model would be different with a higher number of categories. The design matrix of the random terms can be defined by stacking by rows the three matrices $\boldsymbol{Z}_{i,a}=\boldsymbol{I}_{4}$.

**B4. Longitudinal**

For each of the 108 US cities the study interval (1987-2000) was split into five periods: [1987, 1989], [1990, 1992], [1993, 1995], [1996, 1998] and [1999, 2000]. For each period, we fitted distributed lag non-linear time-series models during summer months (June to September). The analysis follows the same scheme as in Section B1, only repeated by period. Thus, we obtained 540 sets of coefficients and associated (co)variance matrix describing the period-specific temperature mortality association nested within 108 cities.

Here ${\hat{\boldsymbol{\theta}}}_{i}$ is a $(20\times1)$ vector (four splines coefficients estimated in the five periods) and the within location (co)variance matrix $\boldsymbol{S}_{i}=\oplus_{p}\boldsymbol{S}_{i,p}$ is a block diagonal matrix where diagonal blocks $\boldsymbol{S}_{i,p}$ are the (co)variance matrices of the coefficients calculated in each period (p).

In addition, we reconstructed location-specific AC air conditioning trends and assigned AC prevalence estimates to each location/period unit (4).

We modelled AC prevalence and calendar year as fixed-effects variables by stacking by rows the matrices $\boldsymbol{X}_{i,p}$, with $\boldsymbol{X}_{i,p}=\boldsymbol{x}_{i,p}^{T}\otimes\boldsymbol{I}_{4}$ , where $\boldsymbol{x}_{i,p}^{T}$ is the vector representing the AC prevalence (linear term) and calendar year (natural spline with one internal knot) in period (p). The design matrix of the random terms can be defined by stacking by rows the five matrices $\boldsymbol{Z}_{i,p}=\boldsymbol{I}_{4}$.

**C. Backward or step-forward procedures**

The analyst could use different procedures to select relevant predictors (e.g. backward or forward stepwise). The backward selection approach starts from the full model (with the 3 predictors), and a likelihood ratio test is performed to compare the full model and the models without each predictor. The step-forward approach starts instead from the model containing only the intercept (Model 0), and at each step the predictor with highest reduction of the Akaike information criterion (AIC) is included.

**References**

1. Bhaskaran K, Gasparrini A, Hajat S, Smeeth L, Armstrong B. Time series regression studies in environmental epidemiology. International Journal of Epidemiology. 2013;42(4):1187-95.

2. Gasparrini A. Modeling exposure-lag-response associations with distributed lag non-linear models. Statistics in Medicine. 2014;33(5):881-99.

3. Gasparrini A, Armstrong B. Reducing and meta-analysing estimates from distributed lag non-linear models. Bmc Medical Research Methodology. 2013;13.

4. Sera F, Hashizume M, Honda Y, Lavigne E, Schwartz J, Zanobetti A, et al. Air Conditioning and Heat-related Mortality A Multi-country Longitudinal Study. Epidemiology. 2020;31(6):779-87.
